# Ground-Level Pruning at Right Time Improves Flower Yield of Old Plantation of *Rosa damascena* Without Compromising the Quality of Essential Oil

**DOI:** 10.3389/fpls.2022.896237

**Published:** 2022-05-17

**Authors:** Shivani, Mitali Mahajan, Babit Kumar Thakur, Probir Kumar Pal

**Affiliations:** ^1^Division of Agrotechnology, Council of Scientific and Industrial Research-Institute of Himalayan Bioresource Technology (CSIR-IHBT), Palampur, India; ^2^Academy of Scientific and Innovative Research (AcSIR), Ghaziabad, India

**Keywords:** essential oil, flower yield, ground-level pruning, β-citronellol, rejuvenation

## Abstract

The essential oil of *Rosa damascena* is extensively used as a key natural ingredient in the perfume and cosmetic industries. However, the productivity and quality of rose oil are a big concern from the old plantation. It is hypothesized that rejuvenation of old rose plantations through ground-level pruning at right time could improve the yield of flowers and the quality of essential oil. Consequently, a field trial was led-out with 10 treatment conditions encompassing two pruning systems (ground-level pruning and ground-level pruning followed by top pruning at the end of December) and five different months of ground-level pruning (June–October) to understand the best pruning practices. In this experiment, the flower yield ranged from 18.32 to 62.40 q ha^−1^, and oil content varied from 0.035 to 0.049% under different pruning systems and months of pruning. Ground-level pruned plants, irrespective of the month, registered statistically (*p* ≤ 0.05) more flower yield (618.62 and 473.29 g bush^−1^) compared with ground-level pruning followed by top pruned plants in both seasons. The average across the pruning system, ground-level pruning in October registered statistically (*p* ≤ 0.05) greater yield of flowers (709.10 and 605.13 g bush^−1^) compared with the ground-level pruning from June to August. Despite significant variations in flower yield among the treatments, the percentage share of the major compounds particularly β-citronellol+nerol and geraniol in the essential oil were not affected (*p* ≥ 0.05) by the pruning month and pruning system. Thus, the finding suggests that the production from the old plantation of *R. damascena* can be improved by ground-level pruning during October under mild-temperate conditions.

## Introduction

Essential oils produced from the plants have various applications in the agriculture, health, food, perfumery, and cosmetics sectors in different ways. There are around 3,000 essential oils that are produced from different plant species. Out of these, about 300 have commercial importance in the flavor and fragrance industries (Van de Braak and Leijten, 1994; Raut and Karuppayil, 2014; Hussain et al., 2016). *Rosa damascena*, commonly known as damask rose, is a most important essential-oil-bearing plant. This species belongs to the family of Rosaceae. Owing to low oil concentration in flowers and the absence of synthetic replacements, the market price of natural rose oil is very high. This crop is commercially cultivated in many countries like Bulgaria, Turkey, Morocco, Iran, Egypt, China, Russia, and India among others. The major uses of rose essential oil are preparations of high-grade perfume and cosmetic products. Thus, the demand for natural rose oil is steadily increasing day by day. In 2018, globally the market size of rose oil was about USD 278.7 million, and it is projected to expand at a CAGR of 6.8% for the prediction period of 2019–2025 (Grand View Research, 2019).

The quality of the essential oil of rose is determined by the proportion share of different groups of compounds like acyclic monoterpenes, aromatic alcohols, and long-chain hydrocarbons. However, the main components of rose oil that define the market value are citronellol, rose oxide, eugenol, geraniol, farnesol, linalool, citronellyl acetate, and methyl eugenol. The essential oil is commonly deposited in the oil ducts, glands, and trichomes of the plants (Baser and Demirci, 2007). The essential oil is extracted by using different methods such as steam distillation, hydrodistillation, or solvent extraction (Pal, 2013). Nonetheless, the quality and recovery of oil depend on prevailing environmental factors during the flowering, genetic as well as agronomic factors including flowering stage, harvesting time, and distillation technique (Baydar and Baydar, 2005; Shawl and Adams, 2009; Najem et al., 2011; Mahajan and Pal, 2020). Additionally, pruning is one type of mechanical stress, which enhances the flower yield of the damask rose. Pruning is an agricultural technique for controlling growth and improving flowers productivity (Sarkka and Erikson, 2003). Moreover, pruning time and types of pruning are important governing factors for deterring the flower yield (Pal et al., 2014; Pal and Mahajan, 2017). In *R. damascena*, the partial pruning system increases flower yield compared with the complete pruning system. The partial pruning system enhances photosynthetic pigment and N content in leaves (Pal and Mahajan, 2017). A partial pruning system is the pruning practice in which a few shoots are left without pruning and the remaining shoots are pruned at a particular height within the same bush (Pal and Mahajan, 2017). In the case of complete pruning, all the shoots within a bush are pruned at a particular height. In another study, it has been observed that pruning at 90 cm height from ground level during the middle of December is ideal in terms of flower yield of 3 years old plantations of *R. damascena* (Pal et al., 2014).

**Figure 1:**
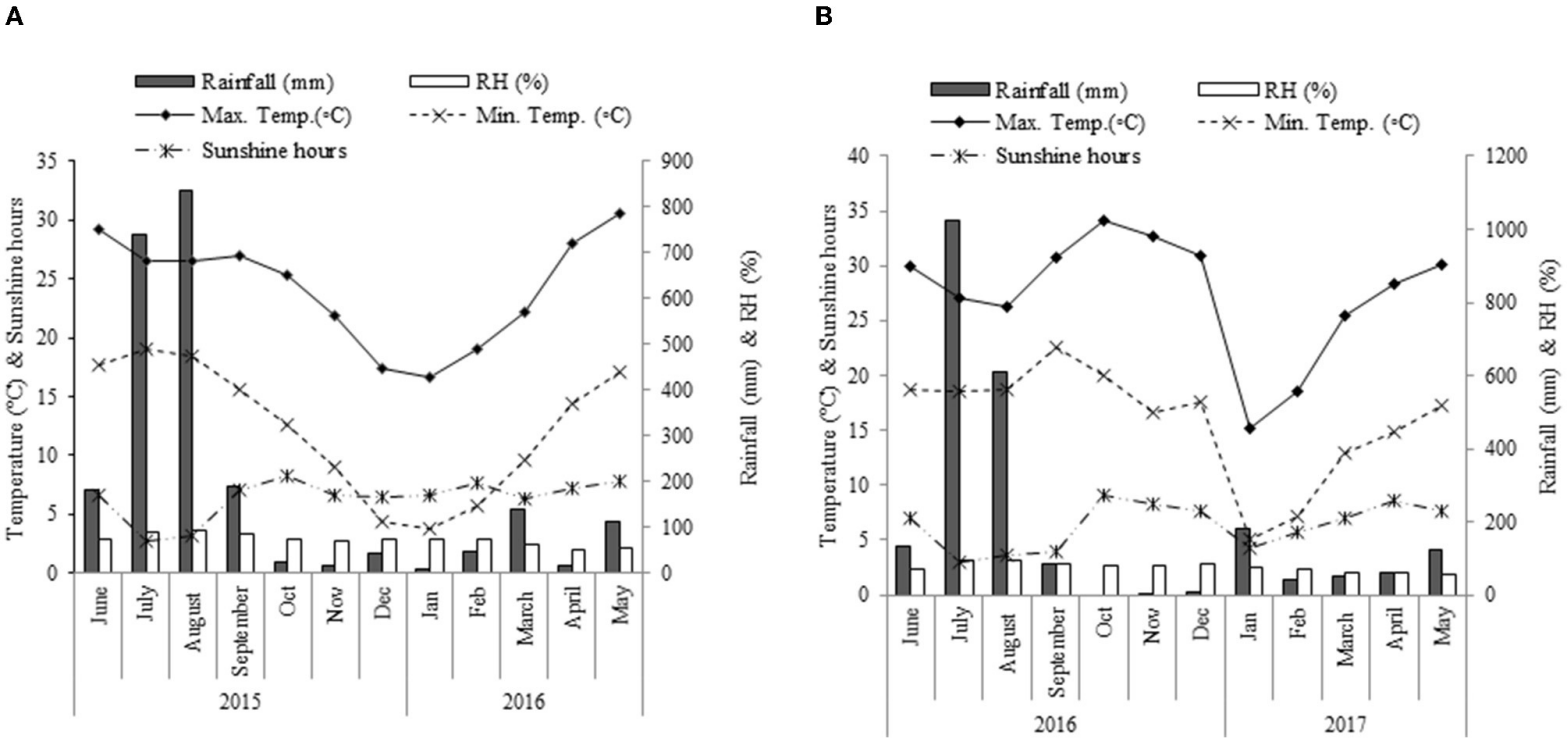
Monthly mean temperatures (°C), sunshine hours (SS), rainfall (mm), and relative humidity (RH) during the cropping season of 2015–2016 **(A)** and 2016–2017 **(B)** at Palampur, India.

The monoculture is mostly practiced in damask rose for maximizing the yield. However, the productivity abruptly declines after 10–12 years of plantation due to depletion of soil health, and abiotic and biotic stress, which may often cause the abandonment of plantations. The overaged bush in old plantations can be rehabilitated by applying different agronomic or horticultural practices. To rejuvenate overaged plantations, rose bushes may be uprooted and re-planted. Nevertheless, this infers an unproductive phase for up to 3 years that is commercially challenging for smallholders. An alternative way to restore higher yield quicker than new planting is the rejuvenation of old bushes by ground-level pruning. In ground-level pruning, all the old shoots are cut at ground level or near ground level. It is hypothesized that in this type of pruning system all the unproductive and dry shoots caused by different biotic and abiotic stresses will be removed, consequently, new vigorous shoots will come out from the crown or roots. An attempt at rehabilitation pruning for cacao (*Theobroma cacao* L.) had been made by Riedel et al. (2019). In the case of damask rose, it is uncertain whether deep-pruning is a feasible approach for the rejuvenation of overaged plantations. Generally pruning operation is done during the dormant phase at a certain height to modify the physiological activities for ensuring fresh axillary bud development (Pal and Mahajan, 2017). In roses, the flowering starts just after the development of axillary bud from pruned stems (Chimonidou et al., 2000). The pruning operation is also practiced to encourage a light reaction, improve metabolic sinks and turgor pressure (Calatayud et al., 2008), and modulate the nutrient cycle (Admasu and Struikb, 2000). Traditional pruning operation (pruned at 60–90 cm height) of damask rose is done during winter (at the dormancy stage) in the western Himalayan region in India (Pal et al., 2014). However, the scientific information on the rehabilitation of overaged damask rose plantation through pruning management is lacking in the mild-temperate hilly region. This lack of scientific knowledge hinders the flower yield from the old plantation of damask rose. It is also hypothesized that the time of ground-level pruning would be another important factor to increase productivity. Moreover, the weather parameters, particularly temperature, humidity, rainfall, and sunshine-hour, have a wide variation from June to October in the western Himalayan region (Figure 1). Thus, this variability insisted we investigate the effects of time of ground-level pruning on flower production and chemical profiling of essential oil of *R. damascena*. Therefore, the aims of this investigation were to (1) study the effect of ground-level pruning on the flower yield of overaged bushes of damask rose; (2) to understand the combined effects of the pruning system and time of ground-level pruning on the yield of flower, essential oil concentration, and quality.

## Materials and Methods

### Experimental Location and Crop Ecology

A field experiment was conducted to understand the influences of ground pruning and time of ground pruning on the flower production, essential oil concentration in flower, and quality of oil of overaged bushes of damask rose during 2015–2016 and 2016–2017. The experiment was conducted at the institutional agricultural farm (CSIR-Institute of Himalayan Bioresource Technology, Palampur) that is positioned at 1,393 m altitude from the mean sea level. The reaction of the experimental soil was acidic with a pH value of 6.2. Organic carbon content was 1.13%. The inherent soil nitrogen (N), phosphorus (P), and potassium (K) contents in the experimental site were also estimated. The available N, P, and K were 260.5, 13.3, and 389.6 kg ha^−1^, respectively, in the top 20 cm soil profile. The main weather parameters during the experimental period are presented in Figure 1.

### Plant Material, Details of the Experiment, and Cultural Practices

In this study, the 10-year-old plantation of *R. damascena* had a plantation geometry of 1.5 m X 0.75 m. The experiment was conducted for two consecutive years. During both years, the crop was fertilized with N, P, and K at 200, 43.7, and 83 kg ha^−1^, respectively. The experiment was conducted under rainfed conditions; thus, additional irrigation water was not applied during the investigation. Other cultural practices were followed as per standard recommendation for *R. damascena* for the western Himalayan conditions. The experiment was performed in a randomized block design (RBD) with the two factors during 2015–2016, and the experiment was again repeated during 2016–2017 cropping season to validate the first-year results. The first factor was the type of pruning with two levels [(i) ground-level pruning (P_1_) and (ii) ground-level pruning followed by top pruning (P_2_)]; the second factor was different months of ground-level pruning [five pruning times i.e. (i) the first week of June, (ii) the first week of July, (iii) the first week of August, (iv), the first week of September, and (v) the first week of October]. Accordingly, the total treatment combinations were 10 (two types of pruning x five pruning times). First, ground pruning was done in the first week of June since the flower appeared up to the first week of May in the western Himalayan conditions. In the case of ground-level pruning followed by top pruning, the top pruning was done at the end of December. In the case of ground-level pruning, plants were pruned one time, while for ground-level pruning followed by top pruning, plants were pruned two times (ground pruning at different months and top pruning during the end of December). All the treatments were replicated three times. Thus, experimental units were 30 (10 treatment combinations x 3 replications). In the case of ground-level pruning, the plants were pruned at 5–8 cm from ground level, whereas only the apical 5–10 cm portion was removed for top pruning.

### Yield Observation

For the recording of treatment-wise yield data, two plants were randomly chosen from all the treatments from all the replications. The chosen plants were marked for the collection of day-to-day data. From the opening date of flower harvesting to the end of flowering, the data on the number of flowers (no. bush^−1^), the weight of individual flowers (g flower^−1^), and the yield of flowers (g bush^−1^) were recorded on a day-to-day basis. The flower yield per hectare (ha) has been expressed in quintal (q) units (1 quintal = 100 kg) throughout the manuscript. The flower harvesting was started on the 9th and 8th of April in 2016 and 2017, respectively, and it was continuing up to 38 days. To prevent the losses of volatile compounds, flowers were harvested in the early morning (6:00–7:30 AM) by manual hand plucking.

### Extraction of Essential Oil

Freshly plucked flowers of damask rose were taken for essential oil extraction. The oil was extracted by the method of hydrodistillation by a Clevenger-type apparatus for 4 h. For distillation, the proportion of flower to water was 1:2 (w/v). These distillation conditions were the same as those earlier reported (Pal and Mahajan, 2017). For extraction of oil, 1 kg of fresh flowers was used for each sample. The quantity of oil extracted from fresh rose petals was recorded and collected in a glass vial. The collected oil samples were kept at 4°C in a dark place for further qualitative analysis. To ensure they were water-free, the oil samples were passed through anhydrous sodium sulfate (Merck). The essential oil content in flowers collected from the different treatments was expressed as a percentage based on a fresh weight basis.

### Gas Chromatographic-Mass Spectroscopic (GC-MS) Analysis

The gas chromatographic and mass spectroscopic (GC-MS) analysis of the rose oil extracted from fresh petals of *Rosa damascena* was executed by a QP2010 GC-MS system (Shimadzu, Tokyo, Japan) which was coupled with an AOC-5000 auto-injector, and DB-5 (SGE International, Ringwood, Australia) fused silica capillary column (30 m × 0.25 mm and a film thickness 0.25 μm). The programmed temperature was from 70 to 220°C (4 and 5 min) with a stepwise increase in temperature at the rate of 4°C min^−1^ for 5 min; the injector temperature was 240°C and interface temperature was 250°C, respectively. The ionization voltage used was 70 eV with 800–50 amu acquisition mass range, and the carrier gas used was helium, whose flow rate was 1.1 ml per min. Homologous series of n-alkanes (C8–C24) was used to calculate the retention indices (RI) of all volatile components. Then, for the identification of rose oil compounds, the calculated values of retention indices (RI) and their mass spectra were matched with the database of the NIST-MS library (Stein, 2005). After that, the quantification was completed by GC analyses.

### Gas Chromatographic (GC) Analysis and Quantification

Gas chromatographic (GC) analysis of the rose oil samples was performed by a Shimadzu GC-2010 gas chromatograph (Tokyo, Japan) which is equipped with a flame ionization detector (FID) and DB-5 capillary column (30 m × 0.25 mm, fused silica, and film thickness 0.25 μm). The operating conditions, particularly, the temperature was set from 70°C (4 min) to 220°C with a stepwise increase in temperature at the rate of 4°C min^−1^ and held for 5 min; the injector temperatures were 240 and 250°C, respectively. Nitrogen was used as the carrier gas with a velocity of 1.05 ml min^−1^. The quantification of compounds was done through peak area normalization, and the response factor was fixed equal to one for each identified component.

### Statistical Analysis

The relevant data collected from this experiment for consecutive 2 years (2015–2016 and 2016–2017) were subjected to the analysis of variance (ANOVA) by Statistica 7 software to test the effects of the treatment. A two-factorial RBD was adopted in this experiment. The treatment means were differentiated with the help of LSD (least significant difference) value at *p* = 0.05. Statistica 7 software was further used to develop a correlation matrix to establish the relationships among the yield and its attributes and different compounds of essential oil. Principal component analysis (PCA) was also executed to appraise the influences of treatments on the chemical constituents of *R. damascena* essential oil and for grouping the treatment.

## Results and Discussion

### Growth and Yield Data

The flower yield of *R. damascena* in per unit area is largely governed by the number of flowers per bush and individual flower weight. In this experiment, the number of flowers per bush was significantly (*p* ≤ 0.05) changed by the pruning types and month of pruning (Table 1). Irrespective of the month of pruning, the ground-level pruning significantly increased the flowers (no. bush^−1^) by about 22 and 27% compared with ground-level followed by top pruning in 2015–2016 and 2016–2017, respectively (Table 1). In the case of flower weight, the results were not consistent over the years. In the second year, the flower weight was significantly reduced with ground-level followed by the top pruning system. In the first year, the flower weight (g flower^−1^) was equal with both the pruning systems (Table 1). However, the flower yield per bush was significantly (*p* ≤ 0.05) increased with the ground-level pruning by about 21 and 34% compared with ground-level followed by top pruning in 2015–2016 and 2016–2017, respectively. Because of the higher number of flowers per bush and individual bush yield, the significantly (*p* ≤ 0.05) higher flower yield (54.44 and 41.65 q ha^−1^) was recorded with the ground-level pruning compared with ground-level followed by top pruning in both years (44.94 and 30.88 q ha^−1^). The low flower yield with double pruning (ground-level followed by top pruning) system may be because the plant was not able to store enough metabolic food for future use or not ready for second mechanical stress in form of pruning after ground-level pruning. The sprouting ability in pruned plants may be clarified as compensatory growth capacity due to the “coordination theory” (Génard et al., 2008). Pruning intensifies leafy shoot growth by altering the shoot/root ratio (Suchocka et al., 2021). The difference in yield parameters might be because of stored metabolic food in the basal portion and rapid physiologically fresh buds growing vigorously in hard pruned plants resulting in more flowers (Jiao and Grodzinski, 1998; Saffari et al., 2004).

**Table 1:** Yield attributes and flower yield of *Rosa damascena* in response to pruning system (P) and month of pruning (M).

**Pruning system (P)**	**Month of pruning (M)**	**Flower (No. bush^−1^)**		**Weight (g flower ^−1^)**		**Flower yield (g bush^−1^)**		**Flower yield (q ha^−1^)**	
		**2016**	**2017**	**2016**	**2017**	**2016**	**2017**	**2016**	**2017**
Ground	June	92.67	75.67	3.02	2.77	278.28	209.89	24.49	18.47
pruning (P_1_)	July	222.67	95.67	2.79	2.92	620.11	279.52	54.57	24.60
	August	252.00	177.00	2.83	3.07	709.68	540.54	62.45	47.57
	September	257.67	223.67	2.75	2.80	713.57	622.79	62.79	54.81
	October	279.00	246.67	2.77	2.90	771.46	713.72	67.89	62.81
	Average	220.80	163.73	2.83	2.89	618.62	473.29	54.44	41.65
Ground and top	June	109.00	75.33	2.87	2.77	309.55	206.43	27.24	18.17
pruning (P_2_)	July	175.67	90.33	2.73	2.64	479.31	238.40	42.18	20.98
	August	190.33	155.00	2.86	2.67	542.88	411.06	47.77	36.17
	September	202.67	148.00	2.86	2.75	574.99	401.89	50.60	35.37
	October	226.00	178.33	2.86	2.79	646.74	496.54	56.91	43.70
	Average	180.73	129.40	2.83	2.72	510.69	350.86	44.94	30.88
Average across	June	100.83	75.5	2.95	2.78	293.92	208.16	25.86	18.32
pruning system	July	199.17	93	2.76	2.78	549.71	258.96	48.37	22.79
	August	221.17	166	2.84	2.87	626.28	475.80	55.11	41.87
	September	230.17	185.83	2.80	2.77	644.28	512.34	56.70	45.09
	October	252.50	212.5	2.81	2.84	709.10	605.13	62.40	53.25
SEm (±) for pruning system		12.06	4.447	0.02	0.048	14.40	6.43	1.27	0.89
LSD (*P* = 0.05) for pruning system		36.10	13.31	NS	0.145	43.12	19.27	3.79	2.68
SEm (±) for month of pruning		19.06	7.031	0.03	0.077	22.77	10.18	2.00	0.57
LSD (*P* = 0.05) for month of pruning		57.08	21.05	0.11	NS	68.18	30.47	6	1.69
SEm (±) for interaction P x M		26.96	9.94	0.05	0.19	32.20	14.39	2.83	1.25
LSD (*P* = 0.05) for P × M		NS	29.77	NS	NS	96.42	43.09	8.48	3.79

*P, M, and P × M indicate pruning system, month of pruning, and interaction between pruning system and month of pruning, respectively, while LSD, SEm, and NS indicate least significant difference, standard error of the mean, and not-significant, respectively*.

The average across the pruning system, the time of ground-level pruning had a significant (*p* ≤ 0.05) effect on the number of flowers per bush, individual bush yield, and flower yield per unit area (Table 1). In the case of the number of flowers per bush, October pruning had a higher value than the rest of the pruning times during both the study years. October-pruned plants registered about 2.5–2.8 times higher number of flowers than the plants pruned during June (lowest value). In the case of flower weight, the effect of time of ground-level pruning was relatively inconsistent over the years.

The effects of ground-level pruning on flower yield were found significant (*p* ≤ 0.05), and the October pruning (just before winter) resulted in the greatest flower yield (62.40 and 53.25 q ha^−1^). The lowest flower yields (25.86 and 18.32 q ha^−1^) were registered with June pruning (just after completion of the reproductive phase). This low yield with early ground-level pruning (June) was probably because the stored metabolic food was utilized for vegetative growth before winter dormancy.

Moreover, non-structural carbohydrates in the stem are declined as a new flash of rose flower shoot initiated as the stored carbohydrates are utilized in a succeeding crop cycle (Kool et al., 1996).

The results of our investigation support the findings of others (Mortensen and Gislerod, 1994) in suggesting that hard pruning in July decreased the yield and stem length of flowers. Ranganathan (2017) also reported that pruning should not be done in tea after a heavy crop. In contrast, the higher flower yield with late ground-level pruning (October just before winter) was probably due to the fact the longer time available to store the metabolic sinks in basal portion and better root development.

Analysis of variance for flower yield also revealed a significant interaction (*p* ≤ 0.05) between the pruning system and the month of pruning, and the utmost yields (67.89 and 62.81 q ha^−1^) were registered with ground-level pruning during October (Table 1). Therefore, only single ground-level pruning during October might be a favorable agronomic intervention to rejuvenate and sustain the flower yield of the damask rose. In this study, the goal is to improve productivity through restoration pruning beyond the average reference yield reported from the same location for the same cultivar (Kaul et al., 1999). Thus, after ground-level pruning of damask rose, irrespective of the month, the second pruning is not feasible, particularly in mild-temperate conditions.

### Oil Content (%) and Oil Yield (kg ha^–1^)

**Figure 2:**
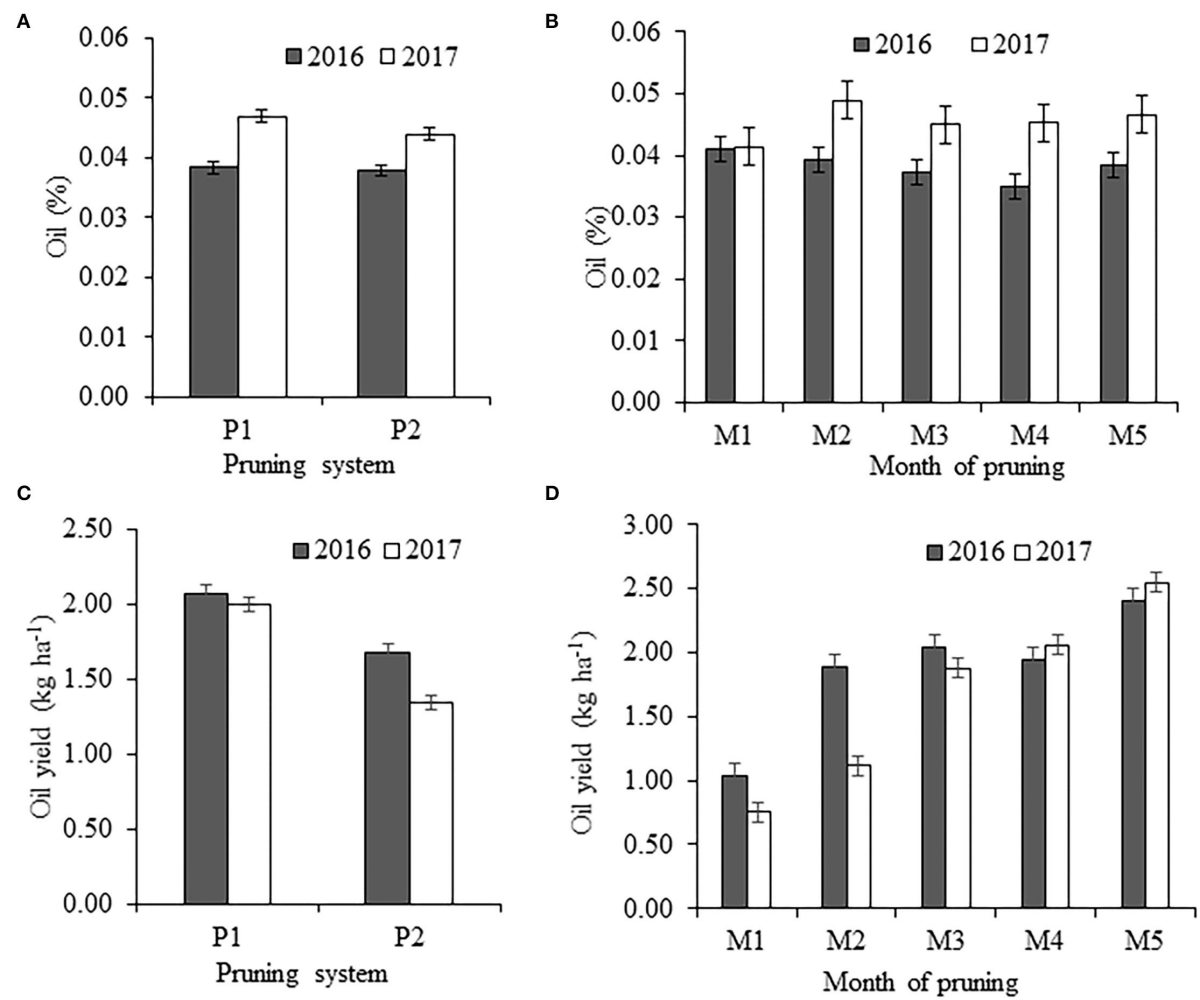
Effect of pruning system and month of ground-level pruning on the oil content (%) in flower **(A,B)** and oil yield (kg ha^−1^) **(C,D)** during the years 2015–2016 and 2016–2017, and the vertical bars indicate mean standard error.

Despite significant variations in flower yields, the concentrations (%) of essential oil in flowers were not significantly (*p* ≥ 0.05) changed during both years (Figures 2A,B). However, irrespective of treatments, the overall oil concentrations in the flower were slightly higher in the second year, and this result may be due to lower flower yield. Thus, these results propose that a combination of two or three management practices to improve flower yield and oil concentration of damask rose may be challenging to achieve. Ground-level pruning in October, irrespective of the pruning system, registered maximum flower yield, but it resulted in a relatively low oil concentration in the first year. It is also a fact that the essential oil yield (kg ha^−1^) was changed significantly (*p* ≤ 0.05) due to different pruning systems and months of pruning (Figures 2C,D). Averaged across the month of ground-level pruning, essential oil yields were about 29 and 47% greater with ground-level pruning than ground-level followed by top pruning in 2015–2016 and 2016–2017, respectively (Figure 2C). This greater essential oil yield with only ground-level pruning was due to higher flower yield without a reduction of the concentration of oil in the flower. Biosynthesis and content of essential oil are largely influenced by environmental factors like temperature and humidity at the time of flowering (Lawrence, 1986; Najem et al., 2011) rather than management factors before flowering. The effect of time of ground-level pruning on the essential oil yield is more pronounced than pruning practices. Irrespective of pruning practices, the plants pruned during October registered about 1.2–2.3 times and 1.2–3.4 times greater essential oil yields in 2015–2016 and 2016–2017, respectively, than the plants pruned in other months (Figure 2D). This improvement in essential oil yield was only due to higher flower yield without losing the content of essential oil.

### Correlations Among Yield and Yield Attributes

The analyses data revealed that the flower yield (q ha^−1^) was positively correlated with the flower number (No. bush^−1^) with *r* values of 1.00 (*p* ≤ 0.01) and 0.99 (*p* ≤ 0.01) in the first and second years, respectively (Table 2). The robust correlation between the flower yield and number of flowers per bush compared to flower weight (r = 0.64; *p* ≤ 0.05 and 0.42) suggests that the number of flowers per bush may be a more important yield attribute for affecting overall flower yield. The absolute positive correlation (*r* = 1.00) was found between flower yield (q ha^−1^) and flower yield per bush. The essential oil concentration was weakly correlated with flower weight, the number of flowers per bush, and flower yield. However, the oil yield (kg ha^−1^) exhibited a strong and positive correlation with the number of flowers per bush (0.98 and 0.97; *p* ≤ 0.01) and flower yield (0.98 and 0.98; *p* ≤ 0.01) as reported by Pal et al. (2016). This result suggests that a higher essential oil yield can be achieved by improving the flower yield through agronomic interventions.

**Table 2:** Correlation matrix among yield components and yield.

**Variable**	**Flower (no. bush^−1^)**		**Flower weight (g flower^−1^)**		**Flower yield (g bush^−1^)**		**Flower yield (q ha^−1^)**		**Oil content in flower (%)**		**Oil yield (kg ha^−1^)**	
	**2016**	**2017**	**2016**	**2017**	**2016**	**2017**	**2016**	**2017**	**2016**	**2017**	**2016**	**2017**
Flower (no. bush^−1^)	1.00	1.00										
Flower weight (g flower^−1^)	−0.68*	0.33	1.00	1.00								
Flower yield (g bush^−1^)	1.00**	0.99**	−0.64*	0.42	1.00	1.00						
Flower yield (q ha^−1^)	1.00**	0.99**	−0.64*	0.42	1.00**	1.00**	1.00	1.00				
Oil content in flower (%)	−0.51	0.37	0.24	0.24	−0.53	0.39	−0.53	0.39	1.00	1.00		
Oil yield (kg ha^−1^)	0.98**	0.97**	−0.65*	0.42	0.98**	0.98**	0.98**	0.98**	−0.35	0.55	1.00	1.00

* Indicates significant at P = 0.05, and ^**^indicates significant at P = 0.01.

*The mean values of the 2-year pooled data of the corresponding treatments are used (where N_1_ = N_2_ = 10)*.

**Figure 3:**
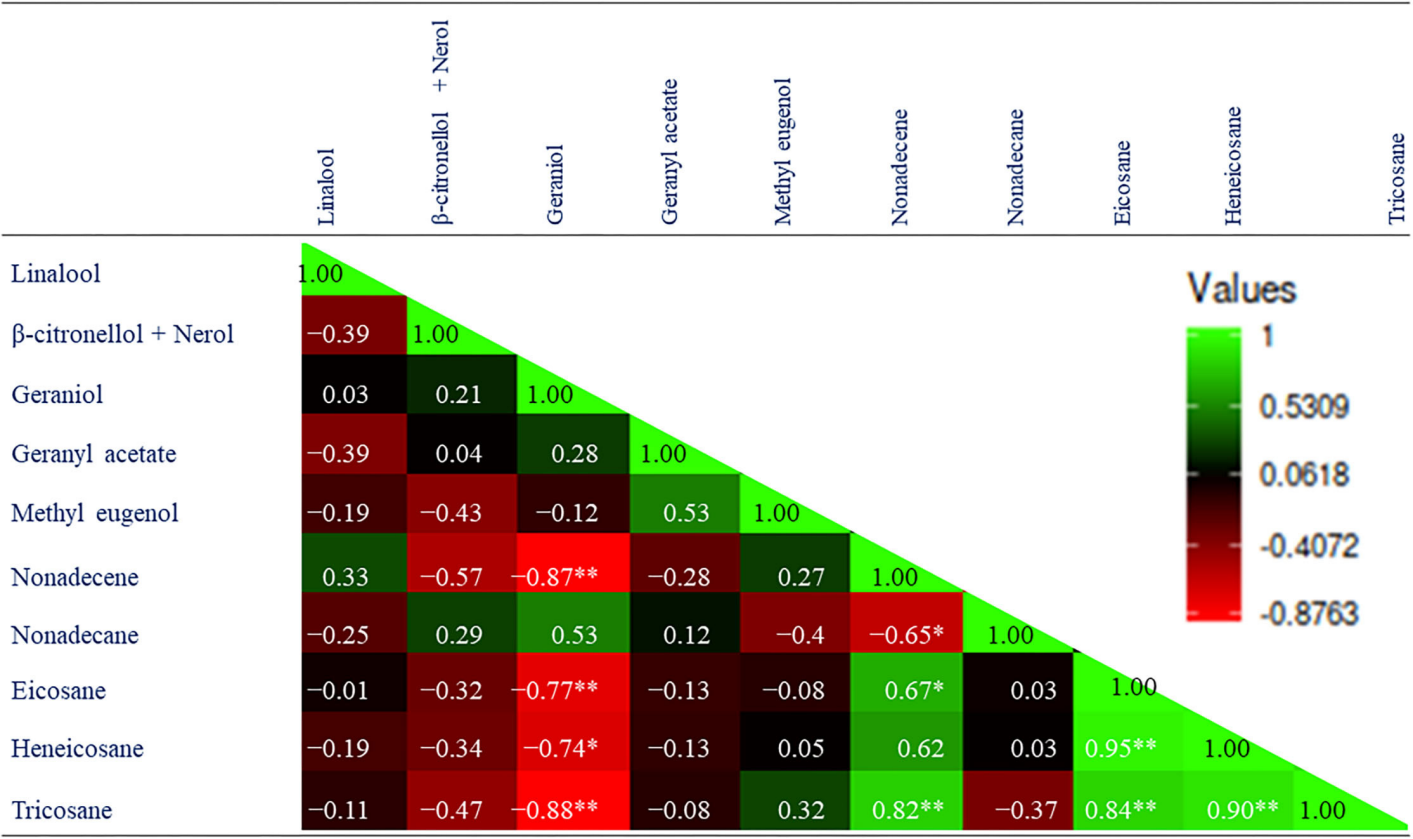
Correlation matrix among the major components of essential oil of *R. damascena*. The mean values of the 2-year pooled data of the corresponding treatments are used (where N1 = N2 = 10).

Although 17 compounds have been identified in the essential oil during both years (Table 3), the correlation matrix has been developed among the 10 major compounds (Figure 3). The results suggest that linalool, β-citronellol + nerol, and methyl eugenol are weakly correlated with each other and with other compounds in the present study. However, geraniol was strong and negatively correlated with nonadecene (r = −0.87; *p* ≤ 0.01), eicosane (r = −0.77; *p* ≤ 0.01), heneicosane (r = −0.74; *p* ≤ 0.05), and tricosane (r = −0.88; *p* ≤ 0.01). The results also suggested that tricosane is strong and positively correlated with nonadecene (r = 0.82; *p* ≤ 0.01), eicosane (r = 0.84; *p* ≤ 0.01), and heneicosane (r = 0.90; *p* ≤ 0.01).

**Table 3:** Variation in essential oil composition of *R. damascena* due to pruning system (P) and month of pruning (M).

**Compounds identified**		**Pruning system (P)**				**Month of pruning (M)**						
	**Year**	**P_1_**	**P_2_**	**SEm (±)**	**LSD (*P* = 0.05)**	**M_1_**	**M_2_**	**M_3_**	**M_4_**	**M_5_**	**SEm (±)**	**LSD (*P* = 0.05)**
Linalool	1st	1.51	1.36	0.08	NS	1.72	1.35	1.40	1.55	1.16	0.13	NS
	2nd	0.82	0.83	0.08	NS	0.93	0.78	0.84	0.69	0.86	0.13	NS
cis-Rose oxide	1st	0.44	0.46	0.02	NS	0.40	0.43	0.43	0.54	0.48	0.03	NS
	2nd	0.36	0.38	0.02	NS	0.44	0.33	0.38	0.33	0.38	0.04	NS
4-Terpineol	1st	0.38	0.38	0.01	NS	0.35	0.35	0.37	0.48	0.36	0.02	0.071
	2nd	0.47	0.47	0.04	NS	0.53	0.44	0.53	0.42	0.43	0.06	NS
β-citronellol+ Nerol	1st	31.26	33.20	1.41	NS	27.70	32.27	32.91	35.20	33.07	2.23	NS
	2nd	37.05	37.45	1.49	NS	37.95	37.89	37.45	34.42	38.54	2.35	NS
Geraniol	1st	18.81	18.42	0.73	NS	20.04	19.16	18.20	17.54	18.15	1.15	NS
	2nd	19.23	21.51	1.11	NS	22.02	20.73	21.15	17.00	20.93	1.75	NS
Eugenol	1st	1.00	0.80	0.05	0.14	1.06	0.74	0.82	1.21	0.67	0.08	0.26
	2nd	0.45	0.50	0.04	NS	0.44	0.42	0.58	0.47	0.45	0.07	NS
Geranyl acetate	1st	2.72	2.22	0.17	NS	2.70	2.54	2.46	2.31	2.35	0.27	NS
	2nd	1.63	1.87	0.22	NS	1.22	1.67	2.06	2.02	1.79	0.34	NS
Methyl eugenol	1st	1.41	1.64	0.05	0.16	1.24	1.43	1.51	1.71	1.73	0.09	0.262
	2nd	0.66	0.70	0.07	NS	0.58	0.64	0.70	0.89	0.59	0.11	NS
α-Humulene	1st	0.59	0.61	0.02	NS	0.53	0.61	0.60	0.63	0.64	0.03	NS
	2nd	0.66	0.72	0.08	NS	0.60	0.72	0.75	0.66	0.73	0.13	NS
Germacrene-D	1st	0.86	0.94	0.04	NS	0.71	0.97	0.89	0.92	1.02	0.07	NS
	2nd	0.70	0.70	0.03	NS	0.72	0.74	0.69	0.57	0.77	0.06	NS
Pentadecane	1st	0.78	0.81	0.03	NS	0.74	0.81	0.81	0.79	0.84	0.05	NS
	2nd	0.59	0.66	0.03	NS	0.64	0.69	0.64	0.59	0.58	0.04	NS
Farnesol	1st	0.99	0.97	0.06	NS	1.12	0.99	0.99	0.87	0.93	0.09	NS
	2nd	1.27	1.27	0.06	NS	1.11	1.31	1.20	1.52	1.21	0.10	NS
Nonadecene	1st	1.80	1.81	0.13	NS	1.97	1.81	1.86	1.57	1.80	0.21	NS
	2nd	3.00	3.35	0.22	NS	3.40	3.44	3.25	3.19	2.59	0.34	NS
Nonadecene	1st	10.68	10.67	0.77	NA	10.84	10.82	11.37	9.84	10.52	1.22	NS
	2nd	12.81	13.74	1.03	NS	13.78	14.26	13.99	10.90	13.44	1.62	NS
Eicosane	1st	1.05	1.01	0.09	NS	0.95	1.04	1.11	1.05	1.00	0.14	NS
	2nd	1.51	1.38	0.13	NS	1.38	1.42	1.49	1.49	1.44	0.21	NS
Heneicosane	1st	4.65	4.82	0.36	NS	4.43	4.82	5.16	4.34	4.92	0.58	NS
	2nd	7.53	7.24	0.60	NS	7.53	7.24	7.34	8.24	7.30	0.96	NS
Tricosane	1st	1.18	1.22	0.12	NS	1.01	1.17	1.30	1.32	1.22	0.20	NS
	2nd	1.73	1.58	0.17	NS	1.44	1.43	1.62	2.13	1.65	0.27	NS

*P_1_ and P_2_ represent the ground-level pruning and ground-level pruning followed by top pruning, respectively, while M_1_, M_2_, M_3_, M_4_, and M_5_ are the ground-level pruning during June, July, August, September, and October, respectively. LSD, SEm, and NS indicate least significant difference, standard error of the mean, and not-significant, respectively*.

### PCA Analysis

**Figure 4:**
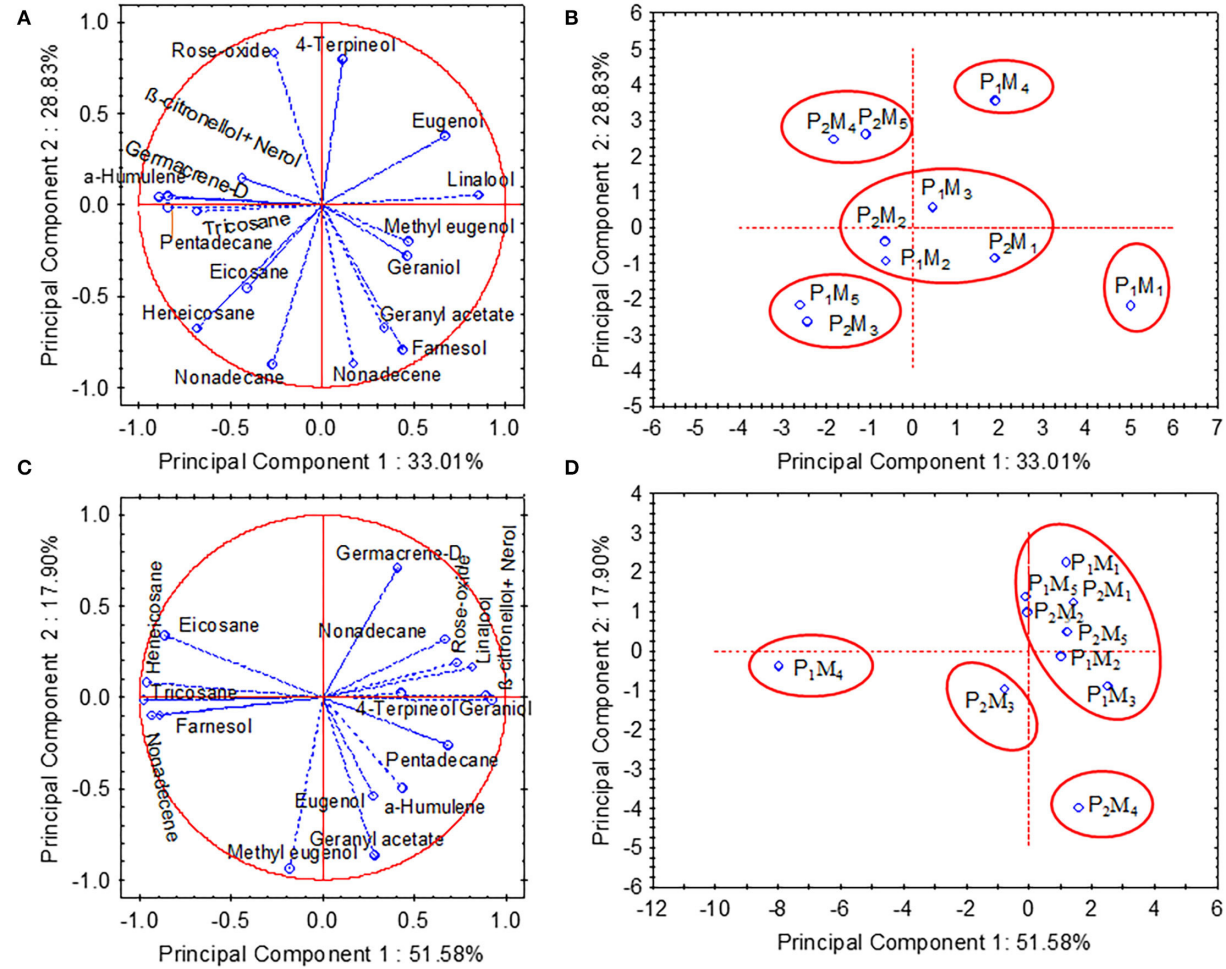
Principal components analysis (PCA) of the components identified in the essential oil of damask rose for the growing seasons of 2015–2016 **(A,B)** and 2016–2017 **(C,D)**. P_1_, Ground-level pruning; P_2_, Ground level-pruning + top pruning; M_1_, Ground-level pruning during June; M_2_, Ground-level pruning during July; M_3_, Ground-level pruning during August; M_4_, Ground-level pruning during September; M_5_, Ground-level pruning during October.

Principal component analysis (PCA) was performed by using 17 compounds of essential oil that were extracted from fresh petals of *R. damascena* in both years. The results from the PCA analysis revealed that the component first and second (PC_1_ and PC_2_) collectively accounted for 61.84 and 69.48% of the total variations for the years 2015–2016 and 2016–2017, respectively (Figures 4A,C). The eigenvalues of the first two most informative principal components are 5.61 and 4.90 in 2015–2016 and 8.77 and 3.04 in 2016–2017, respectively. PCA bi-plot was used to acquire information on treatment combinations (interaction between pruning system and time of ground-level pruning) that were suitable for maintaining the quality of essential oil in this experiment. In 2015–2016, the compounds of essential oil that contributed positively to PC_1_ were linalool, geraniol, eugenol, methyl eugenol, and farnesol with loading values of 0.85, 0.46, 0.67, 0.47, and 0.44, respectively (Figure 4A). In addition, the compounds rose oxide and 4-terpineol contributed more positively to PC_2_ with loading values of 0.84 and 0.80, respectively. The PCA bi-plot also demonstrates that treatments P_1_M_1_ (ground-level pruning during June), P_1_M_3_ (ground-level pruning during August), P_1_M_4_ (ground-level pruning during September), and P_2_M_1_ (ground-level during June followed by top pruning) are separated by PC_1_ from other treatments, and these treatments are in the positive coordinate (Figure 4B). In 2017, farnesol, eicosane, nonadecene, heneicosane, and tricosane developed a distinct cluster, and all these compounds were placed in the negative coordinate of PC_1_ (Figure 4C). On the other hand, linalool, rose oxide, β-citronellol+nerol, geraniol, and nonadecane also developed another cluster with positive loading values of 0.81, 0.73, 0.88, 0.92, 0.67 with PC_1_. Methyl eugenol, a naturally occurring compound in many essential oils, is separated from the rest of the compounds by both PCs and located in the negative coordinate (Figure 4C). For 2016–2017, the PCA bi-plot demonstrates that only treatments P_1_M_4_ (ground-level pruning during September) and P_2_M_4_ (ground-level during September followed by top pruning) are separated from the rest of the treatments (Figure 4D). Thus, the compositions of oil were not largely affected by the interaction of the pruning system and the time of pruning.

### Composition of Essential Oil

**Figure 5:**
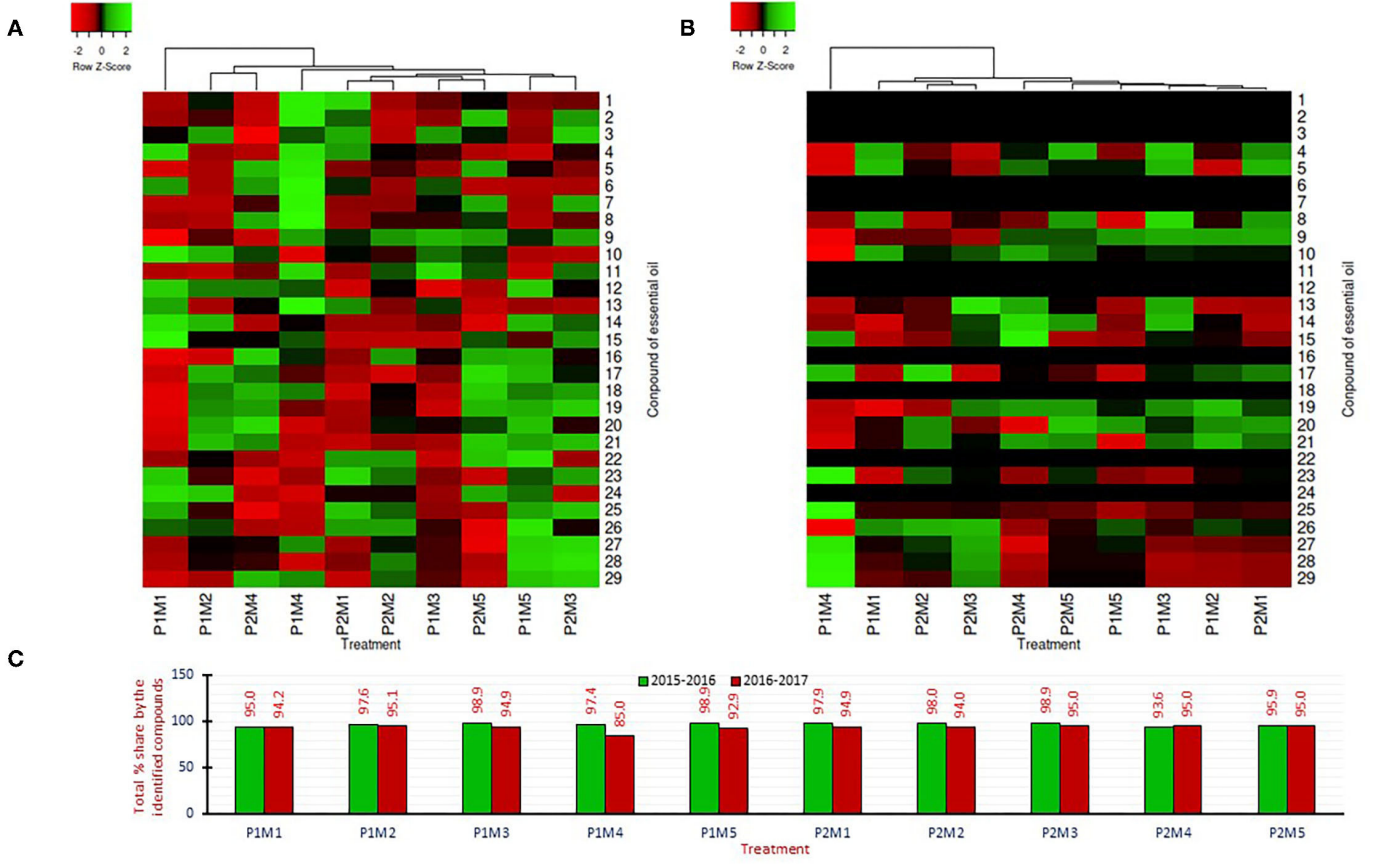
Heatmaps illustrating the variation of components identified in the essential oil of damask rose under different treatment combinations for the growing seasons of 2015–2016 **(A)** and 2016–2017 **(B)**. The total percentage share by the identified compounds is presented in **(C)**. 1, α-Pinene; 2, β-Pinene; 3, β-Myrcene; 4, Linalool; 5, cis-Rose oxide; 6, Phenyl ethyl alcohol; 7, e-Rose oxide; 8, 4-Terpineol; 9, β-Citronellol + Nerol; 10, Geraniol; 11, e-citral; 12, Citronelleyl acetate;13, Eugenol; 14, Geranyl acetate; 15, β-Elemene; 16, Methyl eugenol; 17, E-caryophyllene; 18, α-Guaiene; 19, α-Humulene; 20, Germacrene D; 21, Pentadecane; 22, Heptadecane; 23, Farnesol; 24, Octadecane; 25, Nonadecene; 26, Nonadecane; 27, Eicosane; 28, Heneicosane; 29, Tricosane. P1, Ground-level pruning; P2, Ground level-pruning + top pruning; M1, Ground-level pruning during June; M2, Ground-level pruning during July; M3, Ground-level pruning during August; M4, Ground-level pruning during September; M5, Ground-level pruning during October.

In this study, a total of 29 compounds were identified in both seasons (Figures 5A,B), and the contribution of these compounds in total volume was 85.0–98.9% (Figure 5C). Since the volume contribution is not consistent over the year, it is indicating that climatic conditions during flower harvesting are the possible cause for such variations. The chemical compositions of rose oil in response to interaction effects of the pruning system and month of ground-level pruning are illustrated by heat maps (Figures 5A,B) with *Z*-score. Heat maps describe the changes in the chemical profiling of essential oil. The heat map exhibited that the accumulation patterns of different compounds were not uniform over the years. However, the clustering of treatment in heat maps is like the PC analysis. Thus, to understand the sole effect of individual factors (pruning system and month of ground-level pruning) on chemical compositions, a separate analysis was done (Table 3). In Table 3, the 17 compounds that were identified in all the replications during both years are presented. Across the month of ground-level pruning in the study, the identified compounds (17) of essential oil were not changed significantly (*p* ≥ 0.05) by pruning management, except eugenol in the second year and methyl eugenol in the first year (Table 3). Irrespective of treatment, the content of methyl eugenol in this study was <1.75%, and it does not have a rosaceous quality (Schulz, 2003). Overall, the results somewhat agree with Pal and Mahajan (2017) who reported essential oil composition of the damask rose did not change significantly (*p* ≥ 0.05) by the sole effect of pruning systems like partial and complete pruning.

Average over the pruning system, the compositions of essential oil were not statistically (*p* ≥ 0.05) changed by the time of ground-level pruning, except for 4-terpineol and methyl eugenol in the first year (Table 3). The major compounds, irrespective of treatments, were β-citronellol+nerol (27.70–38.54%), geraniol (17.00–22.02%), nonadecane (9.84–14.26%), and heneicosane (4.34–8.24%). Although the β-citronellol+nerol content was about 26% higher in the September-pruned plants than in the June-pruned plants in 2015–2016, this was not statistically significant (*p* ≥ 0.05). Similarly, the geraniol content was about 12–22% lower in the September-pruned plants than in the June-pruned plants without any statistical differences (*p* ≥ 0.05). The trends of nonadecane and heneicosane contents were irregular, and not similar over the years. However, the overall concentrations of hydrocarbons (nonadecene, eicosane, heneicosane, tricosane) were higher in the second year.

## Conclusions

The most significant finding of this 2-year field experiment is that only ground-level pruning during October can significantly increase flower yield without losing the quantity and quality of essential oil from the old plantation of *R. damascena*. The flower yield was significantly (*p* ≤ 0.05) increased with the ground-level pruning by about 21–34% compared with ground-level followed by top pruning. Among the months of ground-level pruning, October pruning (just before winter) resulted in the utmost flower yield (62.40 and 53.25 q ha^−1^). Similarly, essential oil yields were 29–47% greater with ground-level pruning than ground-level followed by top pruning. Thus, this agronomic strategy is a sensible recommendation, to some extent, for the restoration/ rejuvenation of the low-productive old plantation of *R. damascena*. Additional study is required to elucidate the effects of below-ground competition, plant nutrients, and soil environment on regrowth, the yield of flowers, and the concentration and quality of essential oil of *R. damascena*. The results demonstrated in this investigation were conducted at 1,393 m mean sea level and mild-temperate conditions. The ideal time for ground-level pruning may be different in other environmental conditions.

## Data Availability Statement

The original contributions presented in the study are included in the article/supplementary materials, further inquiries can be directed to the corresponding author.

## Author Contributions

S and MM: data collection, conducting the experiment, essential oil extraction, identification of compounds, and manuscript writing. BT: data collection and essential oil extraction. PP: conceive the ideas, statistical analysis and interpretation, and manuscript writing. All authors contributed to the article and approved the submitted version.

## Funding

This study was completed under project no. HCP-0007 (Aroma Mission), which was funded by CSIR, Government of India.

## Conflict of Interest

The authors declare that the research was conducted in the absence of any commercial or financial relationships that could be construed as a potential conflict of interest.

## Publisher's Note

All claims expressed in this article are solely those of the authors and do not necessarily represent those of their affiliated organizations, or those of the publisher, the editors and the reviewers. Any product that may be evaluated in this article, or claim that may be made by its manufacturer, is not guaranteed or endorsed by the publisher.
